# Members of the Rid protein family have broad imine deaminase activity and can accelerate the *Pseudomonas aeruginosa* D-arginine dehydrogenase (DauA) reaction *in vitro*

**DOI:** 10.1371/journal.pone.0185544

**Published:** 2017-09-28

**Authors:** Kelsey M. Hodge-Hanson, Diana M. Downs

**Affiliations:** Department of Microbiology, University of Georgia, Athens, Georgia, United States of America; East Carolina University Brody School of Medicine, UNITED STATES

## Abstract

The Rid (YjgF/YER057c/UK114) protein family is a group of small, sequence diverse proteins that consists of eight subfamilies. The archetypal RidA subfamily is found in all domains, while the Rid1-7 subfamilies are present only in prokaryotes. Bacterial genomes often encode multiple members of the Rid superfamily. The best characterized member of this protein family, RidA from *Salmonella enterica*, is a deaminase that quenches the reactive metabolite 2-aminoacrylate generated by pyridoxal 5’-phosphate-dependent enzymes and ultimately spares certain enzymes from damage. The accumulation of 2-aminoacrylate can damage enzymes and lead to growth defects in bacteria, plants, and yeast. While all subfamily members have been annotated as imine deaminases based on the RidA characterization, experimental evidence to support this annotation exists for a single protein outside the RidA subfamily. Here we report that six proteins, spanning Rid subfamilies 1–3, deaminate a variety of imine/enamine substrates with differing specific activities. Proteins from the Rid2 and Rid3 subfamilies, but not from the RidA and Rid1 subfamilies deaminated iminoarginine, generated *in situ* by the *Pseudomonas aeruginosa* D-arginine dehydrogenase DauA. These data biochemically distinguished the subfamilies and showed Rid proteins have activity on a metabolite that is physiologically relevant in *Pseudomonas* and other bacteria.

## Introduction

The Rid family is a group of small, sequence diverse proteins that has been divided into eight subfamilies by phylogenetic and bioinformatics analyses [1, 2]. The archetypal RidA subfamily is highly conserved and present in all domains of life, where its importance is reflected by its broad conservation and the diverse consequences caused by its absence. RidA has been implicated in various processes in a number of organisms including bacteria [3–7], plants [8, 9], fungi [10–12], and humans [13–15]. The processes impacted by the relevant RidA member include nutrition [9, 11, 16], mitochondrial maintenance [10–12], development, and carcinogenesis [13, 17, 18]. The *ridA* mutant phenotypes have been mechanistically defined in *Salmonella enterica* where they result from the inactivation of pyridoxal 5’-phosphate (PLP)-dependent enzymes by accumulation of 2-aminoacrylate (2AA) [6, 19–21].

**Fig 1:**
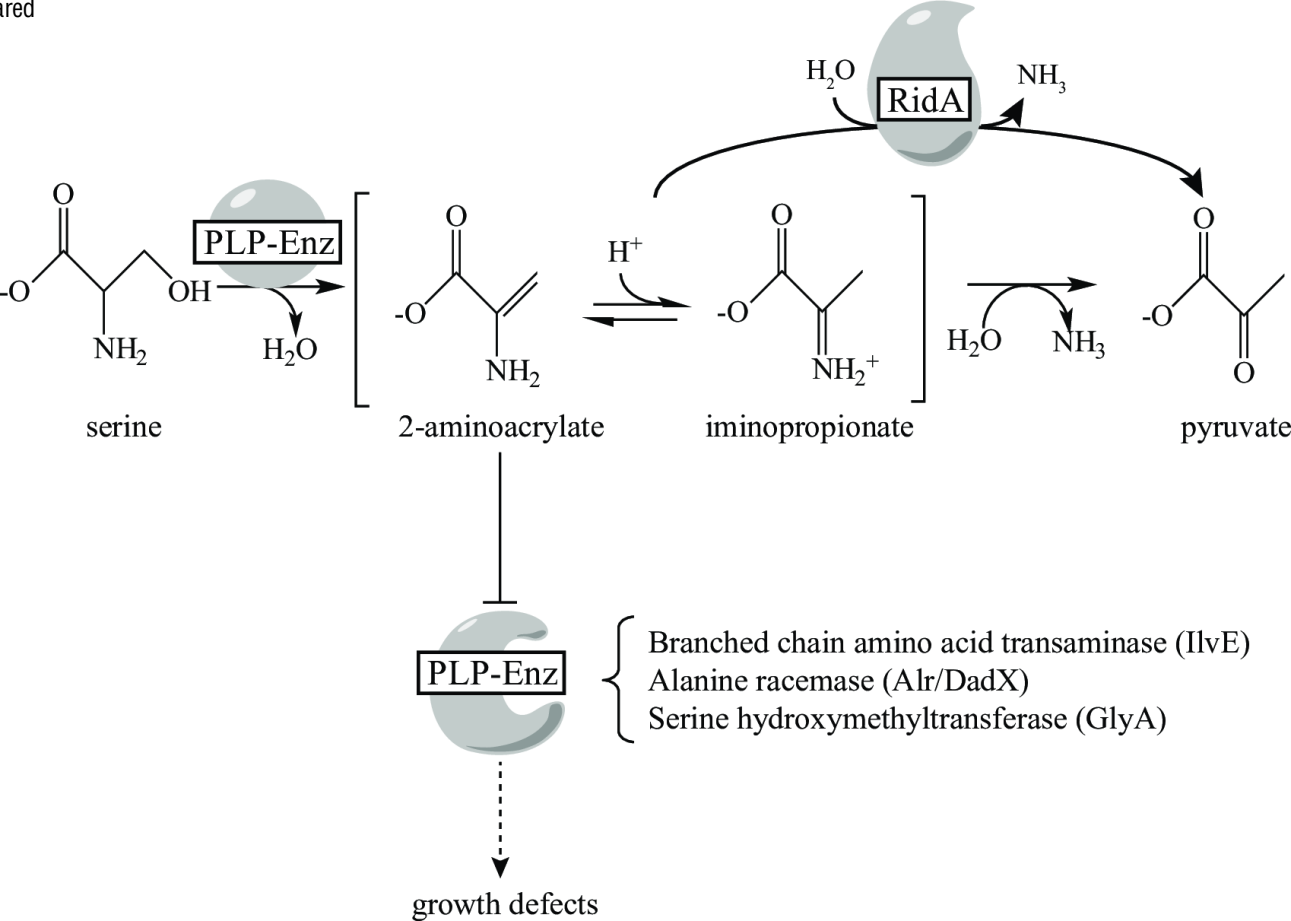
RidA activity *in vivo*. The PLP-dependent generation of the enamine 2 aminoacrylate from serine. Enamine/imine intermediates (2-aminoacrylate/iminopropionate) are in equilibrium and the latter is hydrolyzed by solvent water or facilitated by RidA protein, resulting in production of pyruvate. 2AA accumulation in an *S*. *enterica ridA* mutant is responsible for the inactivation of particular PLP-enzymes, which leads to growth defects.

The biochemical role of RidA has been characterized primarily in the context of specific PLP-dependent enzymes that proceed through 2-aminoacrylate (2AA), which spontaneously tautomerizes in solution to 2-iminopropionate prior to hydrolysis to a stable ketoacid. In the context of PLP-dependent enzymes, RidA increases the rate of 2AA conversion to pyruvate by facilitating hydrolysis, which is otherwise mediated by solvent water (Fig 1) [9, 22, 23]. Members of the RidA subfamily from bacteria, plants, archaea, house dust mite, and humans had activity that was indistinguishable from the *S*. *enterica* protein when tested *in vivo* and *in vitro* [7, 9, 22, 24]. Significantly, high resolution structures of more than twenty RidA homologs had been determined prior to any biochemical information on their function [1, 15, 25–30], many of which exist as Protein Data Bank entries yet to be published.

Unlike the RidA subfamily, which spans the three domains, subfamilies Rid1-Rid7 are found exclusively in prokaryotes [2]. Bacterial genomes often encode multiple members of this protein superfamily suggesting broad, non-redundant involvement in metabolic processes of prokaryotes. For instance, the *S*. *enterica* genome encodes RidA, in addition to YoaB and STM1549, which are members of subfamily Rid2 and Rid7, respectively. The genome of *Escherichia coli* encodes five putative Rid proteins, two of the RidA subfamily, one of the Rid2 subfamily, a Rid7 subfamily protein and RutC [7]. Other representative organisms with numerous Rid family members include *Pseudomonas aeruginosa*, with nine Rid proteins, *Streptomyces coelicolor*, with eleven, and various *Bradyrhizobia* that encode up to sixteen putative Rid proteins [2].

Members of the Rid family are universally annotated as imine deaminases, yet beyond the RidA subfamily, there is a single report of activity for a Rid2 subfamily member [2], and to our knowledge no phenotype has been attributed to the lack of any member of a Rid1-7 subfamily. Based on bioinformatics, protein structures, and activity of RidA variant proteins, the residue analogous to RidA Arg-105 was suggested to be necessary and sufficient to predict deaminase activity [2, 22]. Based on this criterion, Rid1, 2 and 3 subfamily members are expected to have deaminase activity, while subfamilies Rid4, 5, 6, and 7 are not. Thus far, this prediction is supported by a single isolate of Rid2, and a single isolate of Rid7 (YoaB and STM1549 of *S*. *enterica*, respectively) [2].

This study was initiated to validate the broad functional annotation of the Rid superfamily and extend our understanding of the Rid subfamilies beyond the RidA subfamily. Both *in vitro* assays and *in vivo* complementation of a *ridA* mutant were used to assess deaminase activity and substrate specificity of six proteins spanning subfamilies Rid1, Rid2 and Rid3. Iminoarginine deaminase was identified as an activity unique to proteins of the Rid2 and Rid3 subfamilies. In total, these studies bolstered the annotation of the Rid superfamily and suggested a role for Rid family members in facilitating metabolic steps that involve unstable intermediates.

## Materials and methods

### Bacterial strains, plasmids and media

Strains of *Salmonella enterica* were derivatives of serovar Typhimurium LT2 and are described, with relevant plasmids, in Table 1. *Escherichia coli* BL21AI was used for protein overexpression. Primers used to generate plasmids are listed in Table 2. pDM1474 (pBAD24-ACIAD3089) was synthesized by GenScript (Piscataway, NJ) and the coding sequence was codon optimized for expression in *Escherichia coli*. Other plasmids were constructed by amplifying the appropriate gene from genomic DNA, and ligating into a modified pBAD24 vector (pCV1) [31] or into pET28 using BspQI [32]. Plasmid constructs were verified by sequence analysis and sequencing was performed by Genewiz (South Plainfield, NJ) or Eton Bioscience Inc. (San Diego, CA).

**Table 1:** Strains and plasmids.

Strain	Relevant genotype^a^			Encodes	Derivative of
*S*. *enterica* strains^b^					
DM12920	*ridA*1::Tn*10*d(Tc)				pBAD24
DM14846	*ridA*1::Tn*10*d(Tc)		pDM1439	*S*. *enterica* RidA	pBAD24
DM14847	*ridA*1::Tn*10*d(Tc)		pBAD24		pBAD24
DM15406	*ridA*1::Tn*10*d(Tc)		pDM1464	*P*. *aeruginosa* PA0814	pBAD24
DM15407	*ridA*1::Tn*10*d(Tc)		pDM1465	*P*. *fluorescens* PFL_1385	pBAD24
DM15482	*ridA*1::Tn*10*d(Tc)		pDM1474	*A*. *baylyi* ACIAD3089	pBAD24
DM15486	*ridA*1::Tn*10*d(Tc)		pDM1476	*P*. *syringae* PSPTO_0102	pBAD24
DM15440	*ridA*1::Tn*10*d(Tc)		pDM1473	*P*. *syringae* PSPTO_3006	pBAD24
DM15687	*ridA*1::Tn*10*d(Tc)		pDM1515	*P*. *aeruginosa* PA5083	pBAD24
*E*. *coli* strains^c^					
DM12740	pET20b-*ridA*			*S*. *enterica* RidA	pET20b
DM15324	pDM1459			*P*. *aeruginosa* PA0814	pET28
DM15325	pDM1460			*P*. *fluorescens* PFL_1385	pET28
DM15488	pDM1478			*P*. *syringae* PSPTO_3006	pET28
DM15489	pDM1477			*P*. *syringae* PSPTO_0102	pET28
DM15564	pDM1488			*A*. *baylyi* ACIAD3089	pET28
DM15625	pDM1496			*P*. *aeruginosa* DauA	pET28
DM15686	pDM1514			*P*. *aeruginosa* PA5083	pET28
Plasmid	Description or relative genotype				
pBAD24	pCV1; P_*araBAD*_ expression vector, *bla*^+^ (31)				
pET20b	T7 promoter, C-terminal His_6_ overexpression vector, *bla*^+^ (Novagen)				
pET28	T7 promoter, C-terminal His_6_ overexpression vector, *kan*^+^ (32)				

^a^ Tn*10*d(Tc) refers to the transposition-defective mini-Tn*10*(Tn*10*Δ*16*Δ*17*) [33]

^b^ *Salmonella enterica* strains are derivatives of *Salmonella typhimurium* serovar LT2

^c^ *Escherichia coli* strains are derivatives of BL21AI

**Table 2:** Primers used in plasmid construction.

Primer name/relevant plasmid	Primer sequence
PR501 (pDM1474 F)	NNGCTCTTCNTTCATGGTGAATAGAGATGATGCCTTTCTAA
PR502 (pDM1474 R)	NNGCTCTTCNTTATCACTCCTGCCCATTATTTTG
PR503 (pDM1464 F)	NNGCTCTTCNTTCATGCAGACATCCCCCGCTCCG
PR504 (pDM1464 R)	NNGCTCTTCNTTATCATGGCCGCACCGCCGC
PR505 (pDM1465 F)	NNGCTCTTCNTTCATGTCTTTGAAAAGCACCGTCGTAG
PR506 (pDM1465 R)	NNGCTCTTCNTTATCAGCGCGCTCGGGC
PR507 (pDM1473 F)	NNGCTCTTCNTTCATGAGCGACAGCATTCAACGC
PR508 (pDM1473 R)	NNGCTCTTCNTTATCACGCGGATCTGGCCGCCA
PR511 (pDM1459 F)	NNGCTCTTCNATGCAGACATCCCCCGCTCCG
PR512 (pDM1459 R)	NNGCTCTTCNGTGTGGCCGCACCGCCGC
PR513 (pDM1460 F)	NNGCTCTTCNATGTCTTTGAAAAGCACCGTCGTAG
PR514 (pDM1460 R)	NNGCTCTTCNGTGGCGCGCTCGGGC
PR515 (pDM1478 F)	NNGCTCTTCNATGAGCGACAGCATTCAACGC
PR516 (pDM1478 R)	NNGCTCTTCNGTGCGCGGATCTGGCCGCCA
PR650 (pDM1476 F)	NNGCTCTTCNTTCATGTCAATCCAGCGCCAG
PR651 (pDM1476 R)	NNGCTCTTCNTTATTACGGCAGCGCGGCAATAA
PR652 (pDM1477 F)	NNGCTCTTCNATGTCAATCCAGCGCCAG
PR653 (pDM1477 R)	NNGCTCTTCNGTGCGGCAGCGCGGCAATAA
PR725 (pDM1496 R)	NNGCTCTTCNTTATCAGGGGGACAGGCGGCG
PR726 (pDM1496 F)	NNGCTCTTCNTTCATGATCGAAGCGGATTACCTCGT
PR729 (pDM1515 F)	NNGCTCTTCNTTCATGGAACCGACCCGTATCG
PR730 (pDM1515 R)	NNGCTCTTCNTTATCAGCCCCTGGCCGCCAC
PR731 (pDM1514 F)	NNGCTCTTCNATGGAACCGACCCGTATCGCC
PR732 (pDM1514 R)	NNGCTCTTCNGTGGCCCCTGGCCGCCACCACG

Difco Nutrient Broth (8 g/ liter) with NaCl (5 g/ liter) was used as rich medium for *S*. *enterica* and Lysogeny Broth (LB) was used for *E*. *coli*. Cells were grown in super broth (32 g/ liter tryptone, 20 g/ liter yeast extract, 5 g/ liter NaCl and 0.2 g/ liter NaOH) for protein overexpression. Difco BiTek agar was added (15 g/ liter) for solid medium. No-carbon E medium (NCE) supplemented with MgSO_4_ (1 mM) [34], trace minerals [35] and glycerol (30 mM) was used as minimal medium. When indicated, compounds were added at the following concentrations: serine, 5 mM; cysteine, 250 μM; L-arabinose, 0.2%; ampicillin, 150 mg/ liter; kanamycin, 50 mg/ liter. Unless stated otherwise, chemicals were purchased from Sigma Aldrich (St. Louis, MO). Crystalline L-amino acid oxidase (A9253 Type I, dried venom *Crotalus adamanteus*), D-amino acid oxidase from porcine kidney (A5222) and catalase from bovine liver (C9322) were hydrated and stored at 4°C. Tris base was purchased from Amresco (Solon, OH).

### Growth analysis

Liquid growth analyses were done in a 96-well microtiter plate using a BioTek Elx808 plate reader with continuous shaking (set to slow on the machine). *S*. *enterica* strains grown overnight in rich medium (2 mL) were used to inoculate the indicated minimal medium (1% inoculum). Growth was monitored as optical density (OD) at 650 nm for strains grown in biological triplicate at 37°C with shaking. Growth curves were plotted using GraphPad Prism (version 6.0g).

### Protein purification

Rid proteins were purified from *E*. *coli* strain BL21AI harboring the appropriate pET construct. RidA protein was purified as described previously [36]. Proteins PA0814, ACIAD3089, PA5083, PSPTO_0102, PFL_1385 and PSPTO_3006 were purified based on the RidA protocol with modifications of buffers and time of induction as needed to maximize protein recovery. All proteins were polyhistidine-tagged and were purified by nickel-affinity chromatography. Overnight cultures in LB (10 mL) were used to inoculate two flasks containing super broth (1.5 L) supplemented with kanamycin. Cultures were grown at 37°C with shaking until they reached an OD_650_ between 0.7 and 1.0. Arabinose (0.1% w/v for PFL_1385 or 0.2% w/v for all other proteins) was added to induce T7 RNA polymerase production and cultures were shifted to 22°C (PA5083), 28°C (RidA), 30°C (PA0814, ACIAD3089, PSPTO_0102, or PFL_1385) or 37°C (PSPTO_3006) for 18 hours. Cells were harvested by centrifugation at 7,000 x g and the pellets were stored at -80°C until use. Binding buffer (50 mM potassium phosphate pH 7.5, 100 mM NaCl, 5 mM imidazole, and 10% glycerol) was added to thaw cells (2 mL per gram wet cell weight) along with lysozyme (1 mg/ mL) and DNase (20 Units/ mL), and the cells were lysed with a French pressure cell. The lysate was clarified by centrifugation (40,000 x g for 45 minutes) and passed through a 0.45 μm syringe filter (Argos Technologies) prior to being loaded onto a 5 mL Ni-NTA FPLC column of HisPur resin (ThermoFisher Scientific) on an ÄKTA FPLC equipped with a UV lamp and Frac920 fraction collector. Protein was eluted with a 0–100% gradient of elution buffer (50 mM potassium phosphate pH 7.5, 100 mM NaCl, 500 mM imidazole and 10% glycerol) per the column manufacturer’s instructions (GE Healthcare). Elution of polyhistidine-tagged proteins was monitored by absorbance at 280 nm over the gradient and occurred between 20–80% elution buffer. Fractions containing desired protein (based on UV absorption spectroscopy, SDS-PAGE gel analysis) were pooled and concentrated with a 7,000 molecular weight cut-off protein concentrator (Millipore). The preparations were dialyzed into storage buffer (50 mM potassium phosphate pH 7.5, 10% glycerol) using a PD-10 desalting column (GE Healthcare). Proteins (0.125–2 μg) were subjected to SDS/PAGE and purity was assessed using a Foto/Analyst FX (Fotodyne) imager and TotalLab Quant v11 densitometry software. Purified PFL_1835 was enriched to greater than 90% purity, while all other Rid proteins used in this study were enriched to greater than 98% purity. Protein concentration was quantified using BCA Protein Assay (Thermo Scientific), and the samples were frozen in liquid nitrogen and stored at -80°C.

DauA was partially purified from DM15625, a BL21AI strain harboring pET28-*dauA*. An overnight culture (10 mL) grown in LB containing kanamycin was used to inoculate 1.5 liters of super broth containing kanamycin. A total culture volume of 3 liters was grown at 37°C until the cultures reached an OD_650_ of 0.6. L-arabinose (3 grams) was added to each flask, and the temperature was shifted to 18°C for 16 additional hours. A sample (5 mL) was pelleted, resuspended in buffer (1 mL of 50 mM potassium phosphate pH7.5, 1 mM EDTA), sonicated, and activity confirmed using a dye-based crude extract dehydrogenase assay [37]. Cells were harvested by centrifugation at 7,000 x g and the pellet (24.5 grams) was stored at -80°C. The cell pellet was thawed with bind buffer (20 mM sodium phosphate pH 7.4, 300 mM sodium chloride, 10 mM imidazole) containing 1 mM PMSF, lysozyme (1 mg/ mL) and DNase (20 Units/ mL). Cells were lysed using a French pressure cell and the lysate was clarified using the methods described above. The cell-free extract was loaded onto a HisPur Ni-NTA resin (1 mL, Thermo Scientific, Rockford, IL) gravity column, washed twice with wash buffer (20 mM sodium phosphate pH 7.4, 300 mM sodium chloride, 25 mM imidazole) and eluted with elution buffer (20 mM sodium phosphate pH 7.4, 300 mM sodium chloride, 250 mM imidazole). The protein sample was dialyzed into storage buffer (50 mM potassium phosphate pH 7.5, 10% glycerol), quantified using BCA Protein Assay (Thermo Scientific), and frozen in liquid nitrogen and stored at -80°C. A sample was evaluated for purity using SDS-PAGE analysis as described for Rid protein, and was found to be 60% pure. The DauA-enriched sample generated imines that were derivatized by semicarbazide and detected within the linear range of the spectrophotometer (Spectramax Plus 384, Molecular Devices, Sunnyvale, CA).

### Enzyme assays

All assays were performed at room temperature (~25°C).

### Cysteine desulfhydrase CdsH

A coupled assay using cysteine desulfhydrase-dependent pyruvate formation and the oxidation of NADH by lactate dehydrogenase was used to assess Rid activity as previously described [23]. The assay contained Tris base (100 mM, pH 8), NADH (0.25 mM), pyridoxal 5’-phosphate (30 μM) and pyruvate kinase/lactate dehydrogenase (5 Units). Purified Rid proteins and cysteine desulfhydrase (CdsH) were added at monomeric concentrations of 0.19 μM and 0.27 μM, respectively. Reactions were started by the addition of a freshly prepared L-cysteine stock to a range of final concentrations between 0.1 and 10 mM, and absorbance at 340 nm was measured over time. Rates represent initial velocities obtained in the first 30 seconds following substrate addition and moles of pyruvate formed were calculated using the molar extinction coefficient of NADH at 340 nm (ε = 6,220 M^-1^ cm^-1^).

### L-amino acid oxidase

The LOX-based assay for Rid activity was adapted from a previously described continuous assay [38]. Simply, the imine intermediates were derivatized with semicarbazide resulting in semicarbazone compounds that were detected by absorbance at 248 nm [2]. The assay mixture (100 μL total volume) contained potassium pyrophosphate (50 mM, pH 8.7), neutralized semicarbazide (10 mM), bovine liver catalase (24 Units), L-amino acid oxidase (0.4 μM) and 10 μM Rid protein. Reactions were started with the addition of an L-amino acid or 2-aminobutyrate to the final concentration indicated. Following the addition of substrate, the path length for each well was measured and used along with the molar extinction coefficient for semicarbazone (ε = 10,300 M^-1^ cm^-1^) to calculate the rate of semicarbazone formation.

### D-arginine dehydrogenase DauA

Reaction mixtures contained potassium pyrophosphate (50 mM, pH 8.7), neutralized semicarbazide (10 mM), bovine liver catalase (24 Units), FAD (30 μM), and ~ 1 μM DauA. The reaction was initiated with the addition of D-arginine (1 mM), and the absorbance at 248 nm was monitored for ten minutes. Similar to assays with LOX, the path length was determined for each well, and the molar extinction coefficient for semicarbazone was used to calculate the rate of semicarbazone formation.

## Results and discussion

### Proteins from Rid1, 2 and 3 subfamilies deaminate 2AA *in vitro*

**Fig 2:**
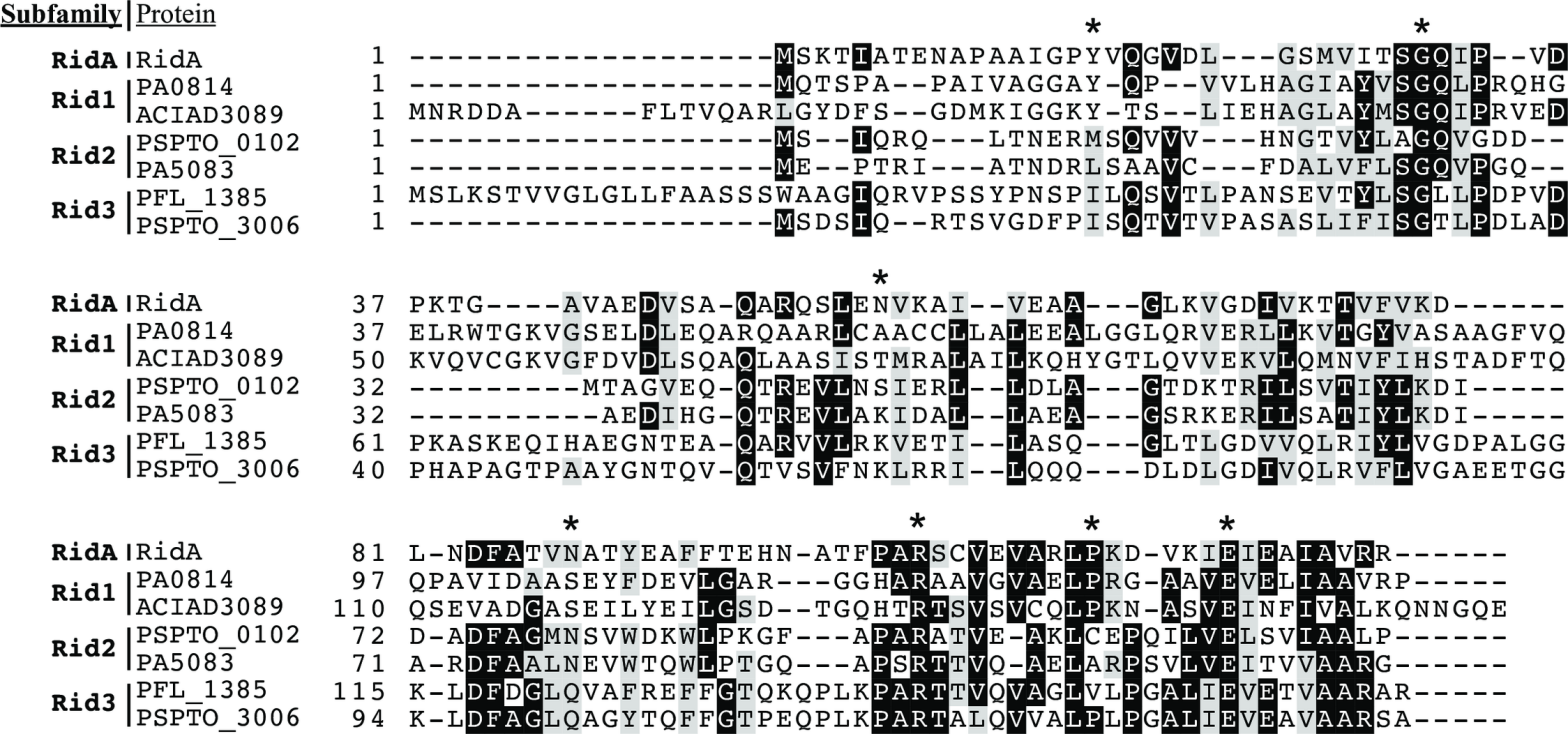
Sequence alignment of Rid proteins. Amino acid sequences of the Rid proteins used in this study are aligned and grouped by subfamily (RidA or Rid1-3). Similar residues are boxed in grey and identical residues are boxed in black. Active site residues proposed for *S*. *enterica* RidA are marked with asterisks. The amino acid sequences were aligned using Clustal Omega [39] and residues were shaded using the ExPASy BoxShade server.

Representative members of Rid1, Rid2, and Rid3 subfamilies, predicted to have deaminase activity due to the presence of the relevant arginine residue, were chosen for study. Rid1 representatives were from *Pseudomonas aeruginosa*, and *Acinetobacter baylyi*. Members of Rid2 came from *S*. *enterica*, *Pseudomonas aeruginosa*, and *Pseudomonas syringae*, and members from Rid3 came from *Pseudomonas syringae* and *Pseudomonas fluorescens*. Source organism, locus tag and sequence alignment of each protein are shown in Fig 2 and Table 3.

**Table 3:** Rid subfamily representatives and identity to *S*. *enterica* RidA.

Subfamily^a^	Locus tag	Organism	Identity (%)^b^	Rid Proteins/genome^c^
Rid1	PA0814	*Pseudomonas aeruginosa* PAO1	26	9
	ACIAD_3089	*Acinetobacter baylyi* ADP1	23	7
Rid2	PSPTO_0102 PA5083	*Pseudomonas syringae* DC3000 *Pseudomonas aeruginosa* PAO1	27 29	8 9
Rid3	PSPTO_3006	*Pseudomonas syringae* DC3000	33	12
	PFL_1385	*Pseudomonas fluorescens* Pf-5	31	8

^a^ Rid subfamily assignments were obtained from the PubSEED database [40].

^b^ Identity (%) to *S*. *enterica* RidA was calculated from the amino acid sequence alignment given in Fig 1.

^c^ For each organism, the total number of Rid proteins encoded in the genome is shown.

**Fig 3:**
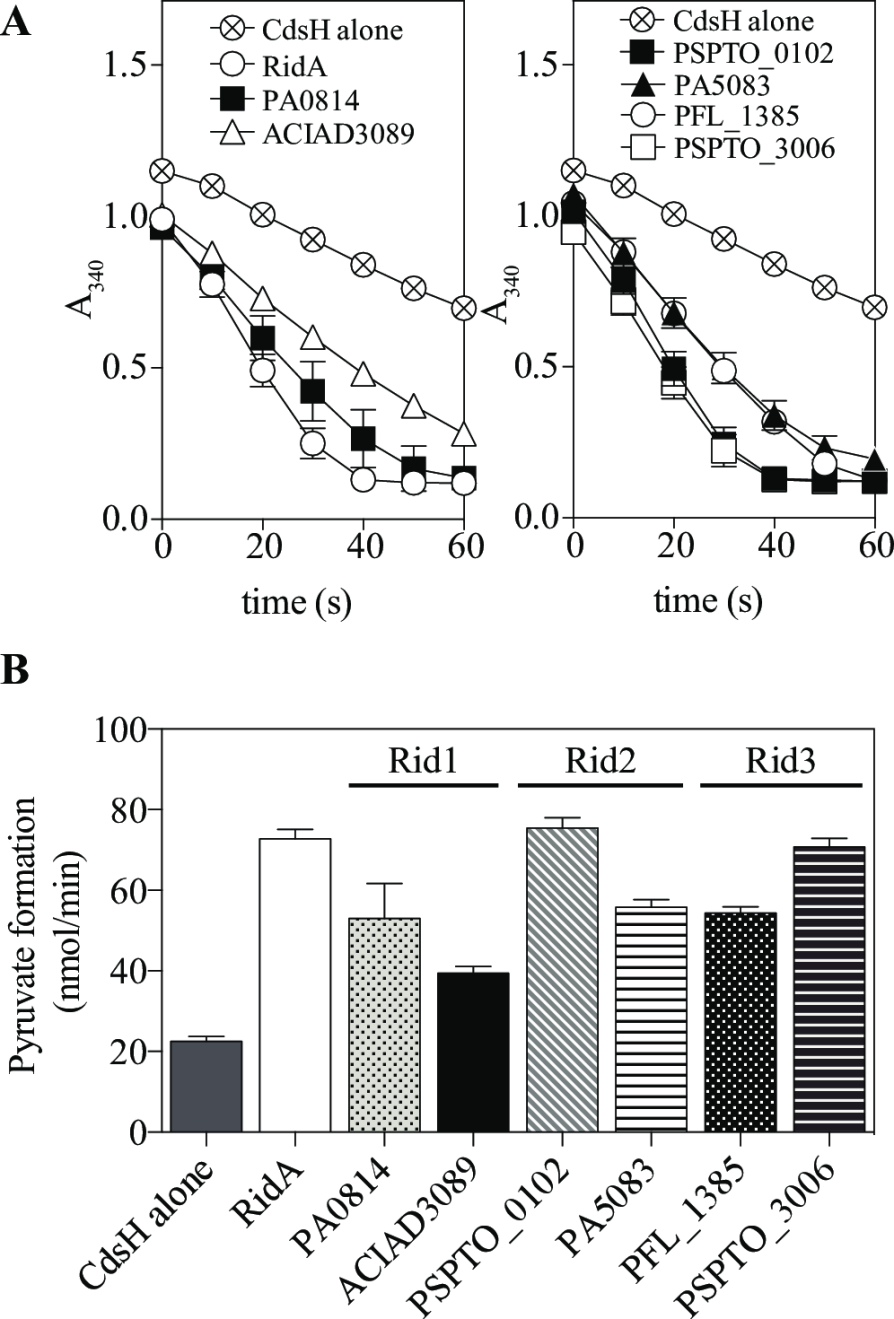
Rid proteins deaminate 2AA *in vitro*. Reaction mixtures (100 μL) contained Tris-HCl (100 mM, pH 8), NADH (250 μM), pyridoxal 5’-phosphate (30 μM), pyruvate kinase/lactate dehydrogenase (5 Units) and purified cysteine desulfhydrase CdsH (0.27 μM). Purified Rid protein (0.19 μM) was added as indicated. **(A)** Absorbance was monitored at 340 nm for 60 seconds following the addition of L-cysteine to 1 mM final concentration. Reactions contained CdsH and purified Rid proteins as indicated. **(B)** The initial rate of pyruvate formation for each reaction was calculated using the molar extinction coefficient (ε = 6,200 M^-1^ cm^-1^) for NADH oxidation during the first 30 seconds. The mean of three replicates is plotted and error bars indicate standard deviation.

Growth defects of a *ridA* mutant have been attributed to the accumulation of 2-aminoacrylate *in vivo* [6, 19–21, 41]. The standard *in vitro* assay for RidA activity uses a PLP-dependent dehydratase to generate 2-aminoacrylate *in situ*. *In situ* generation of the 2AA substrate is necessary due to its short (~1.5 s) half-life in aqueous solution [42]. In this assay, Rid activity is detected as an increased rate of pyruvate formation (from substrate serine) compared to that of a reaction mixture devoid of Rid protein, where deamination is catalyzed solely by solvent water. RidA can deaminate a wide range of substrates, but only 2AA (derived from serine or cysteine) and 2 aminocrotonate (derived from threonine) have been shown to be physiologically relevant [2, 22]. Six Rid proteins (Table 3) were tested for the ability to deaminate 2AA *in vitro* using cysteine desulfhydrase (EC 2.5.1.47, CdsH) as the *in situ* generator. The data in Fig 3 showed all proteins accelerated the rate of pyruvate formation in a coupled assay following NADH oxidation by lactate dehydrogenase. These data indicated that the proteins could deaminate 2AA and strengthened the conclusion that Rid superfamily members with the relevant arginine residue (RidA Arg-105) had deaminase activity.

### Rid proteins functionally complement a *ridA* mutant

**Fig 4:**
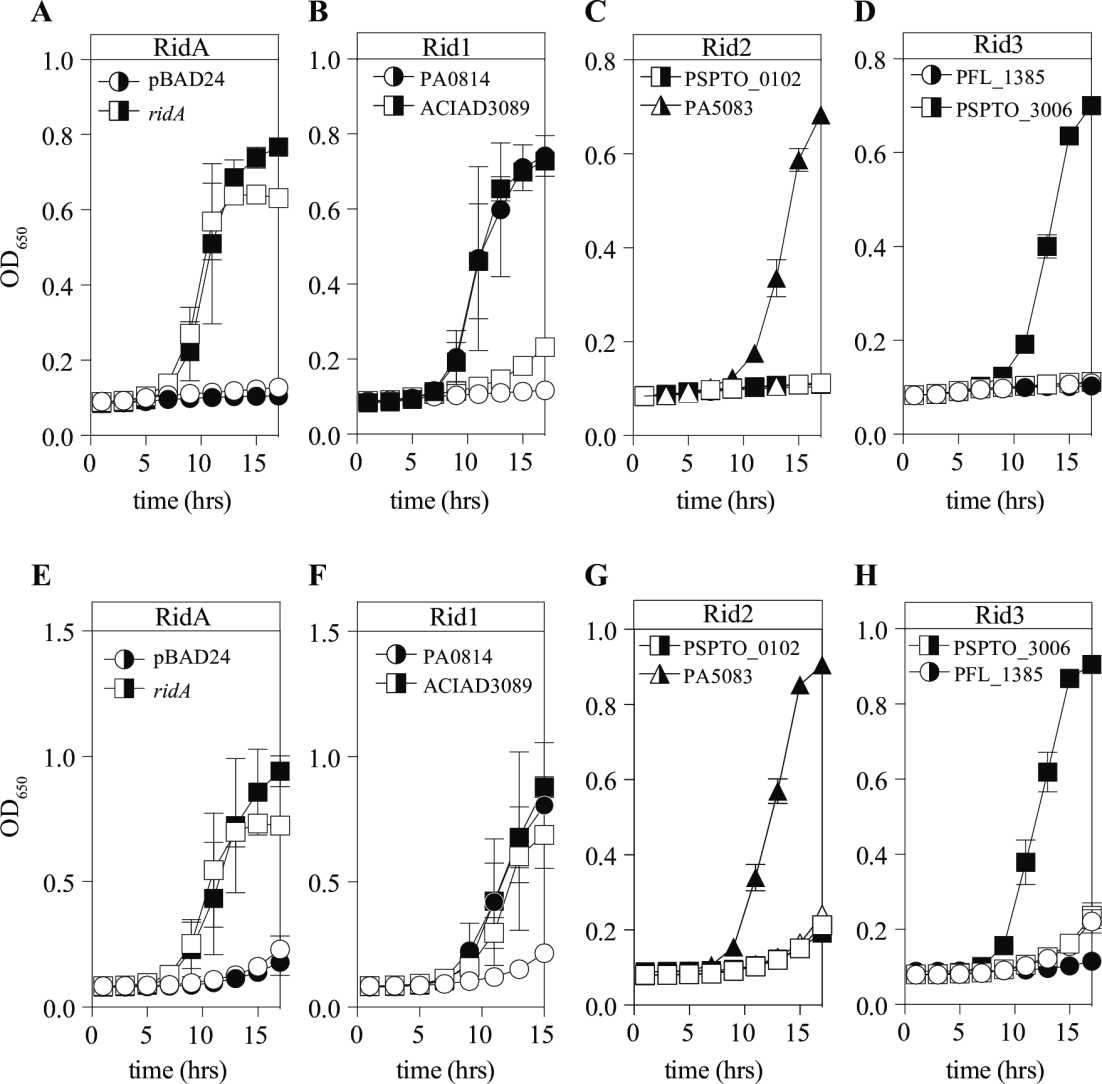
Representative RidA and Rid1 proteins rescue growth of *S*. *enterica ridA* mutant strain. An *S*. *enterica ridA* strain (DM12920) was transformed with pBAD24 constructs harboring no insert (pBAD24), *S*. *enterica ridA*, P. *aeruginosa* PA0814, *A*. *baylyi* ACIAD3089, *P*. *syringae* PSPTO_0102, *P*. *aeruginosa* PA5083, *P*. *fluorescens* PFL_1385, or *P*. *syringae* PSPTO_3006. The strains were grown in minimal glycerol medium supplemented with **(A-D)** 5 mM serine or **(E-H)** 250 μM cysteine, with (closed symbols) or without (open symbols) arabinose. The corresponding Rid subfamily assignment is presented above each graph. Growth was monitored by optical density at 650 nm with shaking at 37°C. Error bars indicate standard deviation for three biological replicates.

*S*. *enterica* strains lacking *ridA* have a growth defect in minimal medium containing 5 mM serine or 250 μM cysteine due to the accumulation of 2AA [16, 23, 43]. These growth defects were used to assess *in vivo* function of representative members of the subfamilies Rid1-3. Rid-protein encoding genes were cloned into pBAD24 vectors, putting their expression under the control of arabinose. Each of the plasmids, in addition to control plasmids pDM1439 and pBAD24, was transformed into strain DM12920 (*ridA*). The resulting strains were grown in minimal medium containing glycerol (30 mM) as the sole carbon source with the addition of serine (Fig 4 panels A-D) or cysteine (Fig 4 panels E-H). Several points were noted in the growth data. First, the control plasmid pDM1439 restored growth in serine and cysteine with and without induction of *ridA* by arabinose (panels A, E). This result indicated that expression of the *ridA* gene from the pBAD plasmid without induction (presumed to be lower than with the other genes with induction), was sufficient to complement the loss of the chromosomal locus. Second, four of the six remaining plasmids supported growth of the *ridA* mutant when the respective genes were induced by arabinose (panels B-D, F-H). Finally, protein ACIAD3089 from *A*. *baylyi* supported growth of the *ridA* mutant, without induction, when cysteine was present (panel F), but required induction to allow growth in the presence of serine (panel B). Significantly, the pattern of complementation did not correlate with subfamily assignment of the proteins. It was formally possible that some (or all) of the negative results *in vivo* were due to instability of the gene products, which was not specifically tested here. However, the ability to purify the relevant proteins in active forms appeared to minimize this possibility.

### Proteins from Rid1, 2, 3 subfamilies have different substrate specificities

**Fig 5:**
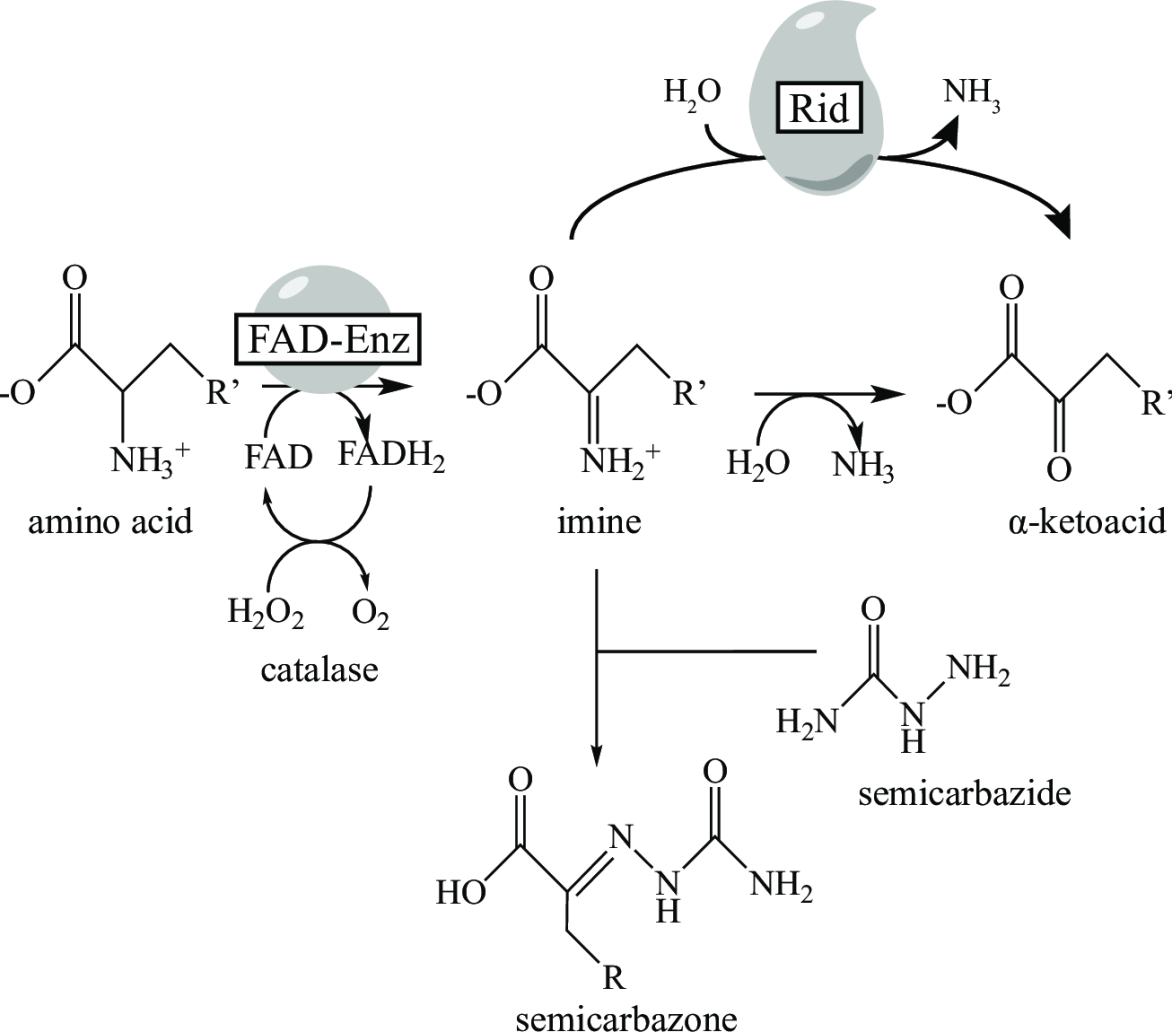
Reaction schemes for FAD-dependent oxidase. The reaction mechanism shows the FAD-dependent production of an imine from an amino acid. Imines are hydrolyzed by solvent water or facilitated by Rid proteins, or in the presence of semicarbazide react to form a semicarbazone compound. R’ represents the amino acid functional group.

Imine deaminase activity of the six Rid proteins was quantified using L-amino acid oxidase (LOX) in an assay described in a previous study, which used proteins RidA, YoaB (Rid2), and STM1549 (Rid7) from *S*. *enterica* [2]. LOX is a promiscuous enzyme that generates imines *in situ* from a range of L-amino acids using an FAD-dependent mechanism. In an assay mixture with Rid proteins included, the resulting imines have three fates that are depicted in Fig 5, i) they can react with semicarbazide to form a semicarbazone that absorbs at 248 nm, ii) they can be converted by solvent water into the corresponding α-ketoacid, or iii) they can be converted by a Rid protein to the α-ketoacid. In this assay, the rate of semicarbazone formation is inversely proportional to Rid activity since the reduction in semicarbazone formation corresponds to an increase in Rid-dependent α-ketoacid formation [9]. Each of the six putative Rid proteins tested slowed the rates of semicarbazone formation in reaction mixtures with a variety of amino acid substrates of LOX as the source of the reactive imine intermediate (Table 4, reaction mixtures 2–8 vs 1). Reaction mixture #1 did not contain a Rid protein and was used to quantify imine derivatization when α-ketoacid formation was mediated only by solvent water. Reaction mixture #2 contained RidA and was used as positive control for activity, defined here as the ability to decrease imines available for derivatization by semicarbazide.

**Table 4:** Rid proteins deaminate a range of imine substrates^a^.

			Rate of semicarbazone formation				
			Substrate				
Reaction	Subfamily	Rid protein	L-Leu	L-Gln	L-Phe	L-Met	L-his
1	-	None	46 ± 0.7	18 ± 0.6	21 ± 0.9	32 ± 0.3	22 ± 2.3
2	RidA	RidA	6 ± 0.4	3 ± 0.2	16 ± 1.3	4 ± 0.3	19 ± 0.1
3	Rid1	PA0814	5 ± 0.8	1 ± 0.3	4 ± 0.7	4 ± 0.4	12 ± 0.3
4		ACIAD3089	9 ± 0.1	4 ± 0.8	14 ± 1.3	6 ± 0.3	18 ± 1.6
5	Rid2	PSPTO_0102	3 ± 0.1	0 ± 0.3	3 ± 0.1	4 ± 0.2	9 ± 0.4
6		PA5083	2 ± 0.4	1 ± 0.4	3 ± 0.9	3 ± 0.2	9 ± 0.3
7	Rid3	PFL_1385	2 ± 0.6	1 ± 0.3	3 ± 0.2	5 ± 0.2	9 ± 0.3
8		PSPTO_3006	2 ± 0.2	0 ± 0.7	3 ± 0.2	7 ± 0.4	8 ± 0.4

^a^ Each reaction mixture (100 μL) contained potassium pyrophosphate (50 mM, pH 8.7), neutralized semicarbazide (10 mM), *Crotalus adamanteus* L-amino acid oxidase (0.4 μM), bovine liver catalase (24 Units) and the indicated Rid protein (10 μM). Reactions were initiated with the indicated amino acid substrate to a concentration of 10 mM. The path length of each well was measured and the absorbance at 248 nm was monitored for five minutes. The rate of semicarbazone formation was calculated from the observed rate using the molar extinction coefficient for semicarbazone (ε = 10,300 M^-1^ cm^-1^). Values represent the average and standard deviation of three replicates (average ± SD). The reactions are numbered in the leftmost column.

In total, the data in Table 4 supported the conclusion that the six proteins annotated as Rid family members had imine deaminase activity on multiple substrates. Beyond this general conclusion, a few observations stood out. First, RidA did not have significant activity with the imines derived from L-Phe and L-His, while the protein was active on the imines of L-Leu, L-Gln and L-Met. Second, the *A*. *baylyi* ACIAD3089 protein had a level of activity similar to RidA across substrates. With a few outliers, the remaining proteins, all from *Pseudomonads*, displayed activity on all substrates similar to or higher than, the RidA protein. A notable observation was that the non-RidA proteins had significantly more activity than RidA with the imine derived from L-Phe and L-His. The significance of this substrate specificity is unclear, but it may hint at a physiological role of Rid proteins.

### Deamination of iminoarginine separates Rid subfamilies

When arginine was used as a LOX substrate, representatives from the Rid1, Rid2 and Rid3 subfamilies appeared to have more deaminase activity than RidA (data not shown). This activity distribution piqued our interest due to the presence of iminoarginine in the metabolism of *Pseudomonas*, the source of the relevant enzymes.

**Fig 6:**
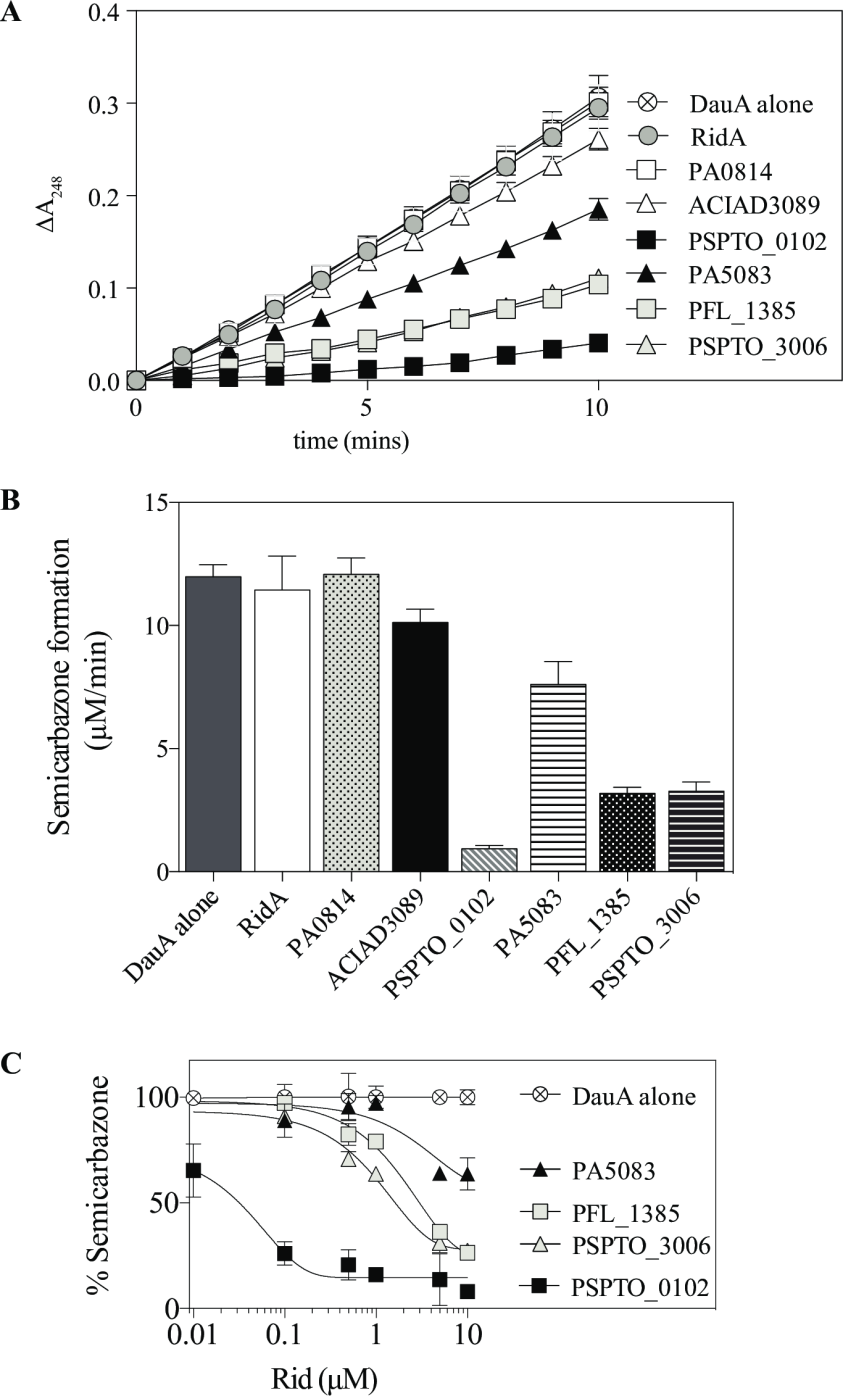
Rid2 and Rid3 proteins deaminate iminoarginine produced by DauA. Assay mixtures (100 μL) were monitored in microtiter plate format and contained potassium pyrophosphate (50 mM, pH 8.7), neutralized semicarbazide (10 mM), bovine liver catalase (24 Units) and ~1 μM DauA (1.7 μM total protein) from the partial purification. **(A)** Rid protein (10 μM) was added and the substrate was D-arginine (1 mM). Following the addition of substrate, the path length of each well was measured and the change in absorbance at 248 nm was monitored for ten minutes. **(B)** The bar graph shows the rate of semicarbazone formation calculated from the observed rate of product formation at 248 nm using the molar extinction coefficient (ε = 10,300 M^-1^ cm^-1^). **(C)** The rate of semicarbazone formation is presented as a percentage of the rate observed in the control (DauA alone) reaction mixture that lacks Rid proteins. All reaction mixtures contained ~ 1 μM DauA and were initiated with 1 mM D-arginine. The effect of purified Rid2 and Rid3 protein concentration on the rate is shown. Error bars indicate standard deviation of three replicates and curves were fitted using the equation for one phase exponential decay using Prism.

In *P*. *aeruginosa*, L-arginine is generated in a racemization pathway that includes D-Arg dehydrogenase (a.k.a. D-Arg:acceptor oxidoreductase (deaminating), EC 1.4.99.6, DauA), to convert D-Arg to 5-guanidino-2-ketopentanoate via an iminoarginine (5-carbamimidamido-2-iminopentanoate) intermediate [44–46]. Given that iminoarginine is an intermediate of the DauA-catalyzed reaction, it was possible that Rid proteins could accelerate the deamination of iminoarginine *in vivo*. Such a scenario would require the release of the iminoarginine from the DauA active site to make it available for the Rid proteins. This possibility was addressed *in vitro* with purified DauA, monitoring semicarbazone formation with a strategy similar to that of the LOX assay. In the absence of a Rid protein, control reactions were initiated with D-Arg (1 mM) and showed the rate of semicarbazone product formation was linear over a DauA protein concentration between 0.3–6 μM (data not shown). The effect of Rid proteins on the rate of semicarbazone formation was determined and the data are reported as a change in absorbance at 248 nm as a function of time (Fig 6A), or as the rate of semicarbazone formation (Fig 6B). As shown in Fig 6, members of the Rid2 subfamily (PSPTO_0102, PA5083) and the Rid3 subfamily (PFL_1385, PSPTO_3006) significantly decreased the rate of semicarbazone formation indicating these proteins used iminoarginine as a substrate. In contrast, neither RidA, nor the Rid1 subfamily proteins PA0814 or ACIAD3089 had significant activity in this assay. These results identified an activity unique to Rid2 and 3 subfamilies. In addition, the results allowed the conclusion that the DauA enzyme released iminoarginine from the active site, where it was then available as substrate for Rid proteins. It is formally possible that the Rid2 and Rid3 proteins are interacting with DauA. Based on RidA precedent and the ability of these Rid proteins to act on iminoarginine produced by eukaryotic enzymes in addition to bacterial DauA, we consider this possibility unlikely. These data defined the third bacterial enzyme mechanism to be associated with Rid function.

In additional assays, arginine concentration was held constant at 1 mM and the concentration of the relevant four Rid proteins was titrated. The data showed that as Rid protein concentration decreased, the rate of semicarbazone formation increased (Fig 6C). These data demonstrated that the fate of the imine depended upon Rid protein concentration, as expected for a process in which the protein was competing with the semicarbazide for the available iminoarginine.

## Conclusions

Results presented here have expanded our understanding of the activities catalyzed by Rid family members beyond RidA. Each of the six proteins studied, representing three Rid subfamilies, had deaminase activity on a range of substrates *in vitro*. These data validated the annotation of the Rid superfamily, and supported the scenario in which the active site arginine residue analogous to RidA Arg-105 was the determinant of deaminase activity.

**Fig 7:**
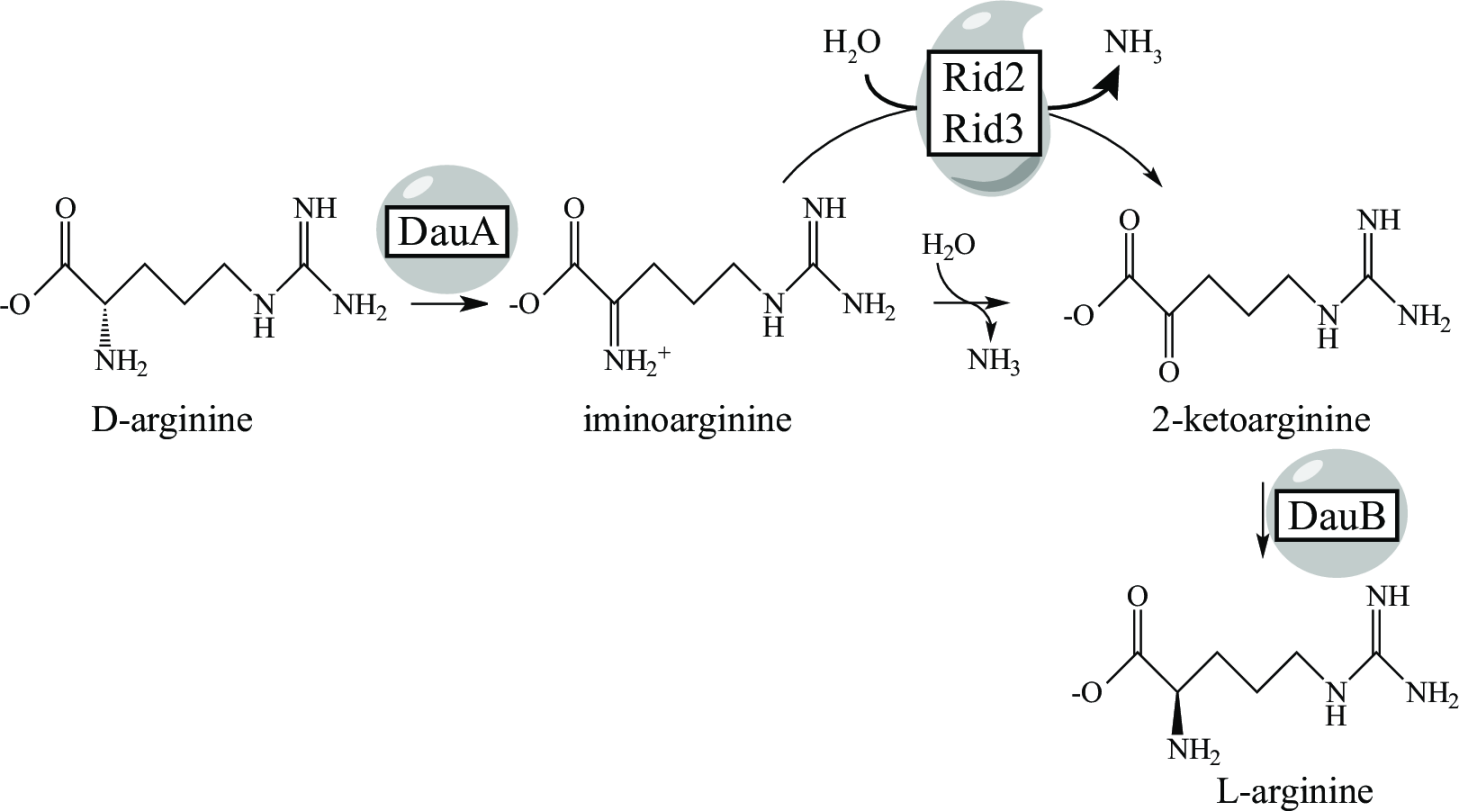
Working model for Rid2 and Rid3 activity. The FAD-dependent D-arginine dehydrogenase generates iminoarginine, which is deaminated by solvent water or by Rid proteins (Rid2 or Rid3), resulting in 2-ketoarginine formation. In *Pseudomonas aeruginosa*, DauB converts 2-ketoarginine to L-arginine, enabling the organism to grow on D-arginine as the sole carbon and nitrogen source [44].

Activity in *in vitro* assays has the caveat that it may or may not represent a physiologically relevant role of the protein. For this reason, the finding that Rid2 and Rid3 subfamilies were active on the iminoarginine generated by the DauA enzyme was significant. First, DauA has a central metabolic role in *Pseudomonas*, the organismal source of several of the Rid proteins tested. Second, the ability of Rid proteins to compete with semicarbazide for the iminoarginine product showed that the imine was released from the DauA enzyme active site. With these data, DauA became the third defined bacterial reaction mechanism to use either a Rid protein or solvent water to generate a ketoacid, the required substrate for a subsequent enzymatic step. This result led to the hypothesis that a Rid protein(s) participates in the pathway of D-arginine racemization *in vivo* (Fig 7). While in a simple scenario, blocking the relevant Rid protein would result in the inability to racemize arginine, this is not likely to be the case. The redundancy of Rid activity with that of solvent water means that Rid proteins facilitate a reaction that occurs, albeit slowly, in the cell. This makes demonstrating an *in vivo* role challenging since loss of the Rid protein is expected to generate a subtle pathway defect, and may not produce a clear phenotype. In fact, the role of RidA was uncovered not because a product was produced more slowly, but because the accumulated substrate (i.e., 2-aminoacrylate) had detrimental effects. We hypothesize that the general role of Rid proteins is to facilitate reactions that are limited by the rate of hydrolysis allowed by water, and thus make a variety of metabolic pathways more efficient. In total, efficiency provided by Rid proteins, and other potential metabolic modulators, would increase overall organismal fitness enough to explain the selective pressure that maintained this superfamily as reflected in its broad conservation.

Despite their role in overall fitness, identifying specific pathways that produce substrates for Rid proteins is hampered by the expectation that any growth defects would be subtle. Defining the physiological roles of Rid proteins will require multiple approaches including creative phenotypic analysis, targeted biochemical approaches and a fair amount of serendipity. The results here provide a framework to explore the role of Rid proteins, specifically in the racemization of arginine in *P*. *aeruginosa*.
